# Molecular surveillance of the *Plasmodium vivax* multidrug resistance 1 gene in Peru between 2006 and 2015

**DOI:** 10.1186/s12936-020-03519-8

**Published:** 2020-12-04

**Authors:** Fredy E. Villena, Jorge L. Maguiña, Meddly L. Santolalla, Edwar Pozo, Carola J. Salas, Julia S. Ampuero, Andres G. Lescano, Danett K. Bishop, Hugo O. Valdivia

**Affiliations:** 1 Department of Parasitology, U.S. Naval Medical Research Unit No, 6 (NAMRU-6), Lima, Peru; 2 Emerge, Emerging Diseases and Climate Change Research Unit, School of Public Health and Administration, Universidad Peruana Cayetano Heredia, Lima, Peru; 3 Departamento de Parasitología, Instituto de Ciências Biológicas, Universidade Federal de Minas Gerais, Belo Horizonte, MG Brazil; 4 Piura Sanitary Intelligence Unit, Piura Health Region, Piura, Peru

**Keywords:** Malaria, *Plasmodium vivax*, Single nucleotide polymorphisms, Drug resistance

## Abstract

**Background:**

The high incidence of *Plasmodium vivax* infections associated with clinical severity and the emergence of chloroquine (CQ) resistance has posed a challenge to control efforts aimed at eliminating this disease. Despite conflicting evidence regarding the role of mutations of *P. vivax* multidrug resistance 1 gene (*pvmdr1*) in drug resistance, this gene can be a tool for molecular surveillance due to its variability and spatial patterns.

**Methods:**

Blood samples were collected from studies conducted between 2006 and 2015 in the Northern and Southern Amazon Basin and the North Coast of Peru. Thick and thin blood smears were prepared for malaria diagnosis by microscopy and PCR was performed for detection of *P. vivax* monoinfections. The *pvmdr1* gene was subsequently sequenced and the genetic data was used for haplotype and diversity analysis.

**Results:**

A total of 550 positive *P. vivax* samples were sequenced; 445 from the Northern Amazon Basin, 48 from the Southern Amazon Basin and 57 from the North Coast. Eight non-synonymous mutations and three synonymous mutations were analysed in 4,395 bp of *pvmdr1*. Amino acid changes at positions 976F and 1076L were detected in the Northern Amazon Basin (12.8%) and the Southern Amazon Basin (4.2%) with fluctuations in the prevalence of both mutations in the Northern Amazon Basin during the course of the study that seemed to correspond with a malaria control programme implemented in the region. A total of 13 *pvmdr1* haplotypes with non-synonymous mutations were estimated in Peru and an overall nucleotide diversity of π = 0.00054. The Northern Amazon Basin was the most diverse region (π = 0.00055) followed by the Southern Amazon and the North Coast (π = 0.00035 and π = 0.00014, respectively).

**Conclusion:**

This study showed a high variability in the frequencies of the 976F and 1076L polymorphisms in the Northern Amazon Basin between 2006 and 2015. The low and heterogeneous diversity of *pvmdr1* found in this study underscores the need for additional research that can elucidate the role of this gene on *P. vivax* drug resistance as well as in vitro and clinical data that can clarify the extend of CQ resistance in Peru.

## Background

In the Americas, *Plasmodium falciparum* and *Plasmodium vivax* are responsible for 25.9% and 74.1% of all malaria cases reported in the region, respectively [1]. Although *P. falciparum* is associated with higher mortality rates, *P. vivax* is attributed with the highest morbidity in the continent and particularly in the Amazon Basin with a ratio of at least 4:1 cases in relation to *P. falciparum* [2].

According to the Peruvian CDC, the Northern Amazon Basin accounted for most of all malaria cases reported in 2018 (Loreto region; 95%) followed by the North Coast (Piura and Tumbes Region; 0.02%) and the Southern Amazon Basin (Madre de Dios; 0.01%). Since 2006, the Northern Amazon Basin experienced a reduction in the incidence of *P. vivax* malaria from 56,171 cases in that year to 26,846 cases in 2010 due to the implementation of the Global fund’s Malaria Project (PAMAFRO), a community programme focused on active case detection and treatment [3]. After the end of the programme in 2010, malaria cases increased each year from 11,779 in 2011 up to 60,268 in 2015.

At the time when samples were collected, there were two treatment schemes for *P. falciparum* that were administered according to the geographical area. In the North coast, standard treatment consisted of sulfadoxine–pyrimethamine (SP) in combination with artesunate (ART), while in the rest of the country it was changed to ART and mefloquine (MQ) as first-line treatment after a brief period of SP [4, 5].

Cases caused by *P. vivax* are primarily treated with CQ and primaquine (PQ) in order to eliminate sexual, asexual and dormant *P. vivax* stages. However, there is accumulating evidence suggesting emergence of *P. vivax* anti-malarial resistance in Peru. For instance, four cases of *P. vivax* recurrence on days 21 and 28 after oral anti-malarial treatment with CQ (10 mg/kg on days 0 and 1 and 5 mg/kg on day 2) and PQ (0.5 mg/kg/day for seven days) were described during a study conducted in 177 patients in the Peruvian Amazon region between 1998 and 2001 [6]. Additionally, Graf et al*.,* reported in 2012 a possible case of chloroquine-resistant *P. vivax* which presented recrudescence on day 28th after treatment with a combination of CQ (25 mg base/kg divided into single daily doses over 3 days) and PQ (0.25 mg/kg daily for 14 days) [7].

Cases of CQ drug resistance in *P. vivax* were first reported in 1989 in Papua New Guinea [8] that subsequently extended to other endemic regions including South America [9–11]. However, the exact mechanisms that modulate drug resistance are still poorly understood. Single nucleotide polymorphisms (SNPs) in *pfmdr1, pfdhps, pfdhfr* and *pfcrt* have been shown to be involved in *P. falciparum* anti-malarial resistance in vivo and in vitro [12–14]. Likewise, mutations on the *P. vivax* orthologous genes *pvmdr1*, *pvdhps*, *pvdhfr* and *pvcrt* have also been associated with anti-malarial resistance although a clear role in treatment failure remains to be fully elucidated [15–22].

In addition, other studies have shown that changes in gene copy number and expression profiles of *pvmdr1* and *pvcrt* could be associated with CQ resistance [23, 24]. The association in the case of *pvcrt* is further supported by recent evidence that 5′UTR and intronic changes in *pvcrt* are linked with increased *pvcrt* expression in parasite CQ resistant cross lines tested in non-human primate models [25]. In the specific case of *pvmdr1*, its role in *P. vivax* resistance is also under discussion in the scientific community with some studies suggesting convergent evolution without an implication in resistance [26–28] whereas others propose a direct association with *P. vivax* resistance [22]. In this regard, studies showed an association between the prevalence of the *pvmdr1* 976F mutation with increased tolerance to CQ and decreased in vitro susceptibility to MQ and ART in areas with high levels of CQ resistance and the opposite in areas where CQ continues to be used as primary treatment [22, 29, 30]. This evidence suggests the presence of competitive evolutionary pressures on *pvmdr1* [29] caused by CQ, MQ and ART and that *pvmdr1* mutations might have significant fitness costs [31].

Many of the initial associations of *pvmdr1* with resistance to CQ were primarily based on molecular tests and epidemiological or clinical information. However, these assessments suffer from important confounding factors [32]. Stronger evidence have been obtained from studies that combined molecular data from *pvmdr1* (SNPs and changes in gene copy number) with in vitro drug susceptibility tests against anti-malarial drugs used in the study area such as CQ and MQ [22, 29] or with clinical data [18, 33].

This study aimed to extend the genetic characterization of *pvmdr1* in South America by assessing changes over time in the prevalence of *pvmdr1* polymorphisms in samples collected across the Amazon basin and other endemic regions from Peru between 2006 and 2015. The information collected will provide a picture of the dynamics of genetic variability of *pvmdr1* in Peru, which can be used as a baseline for surveillance in response to new regional initiatives of malaria control and elimination.

## Methods

### Study design

**Fig. 1:**
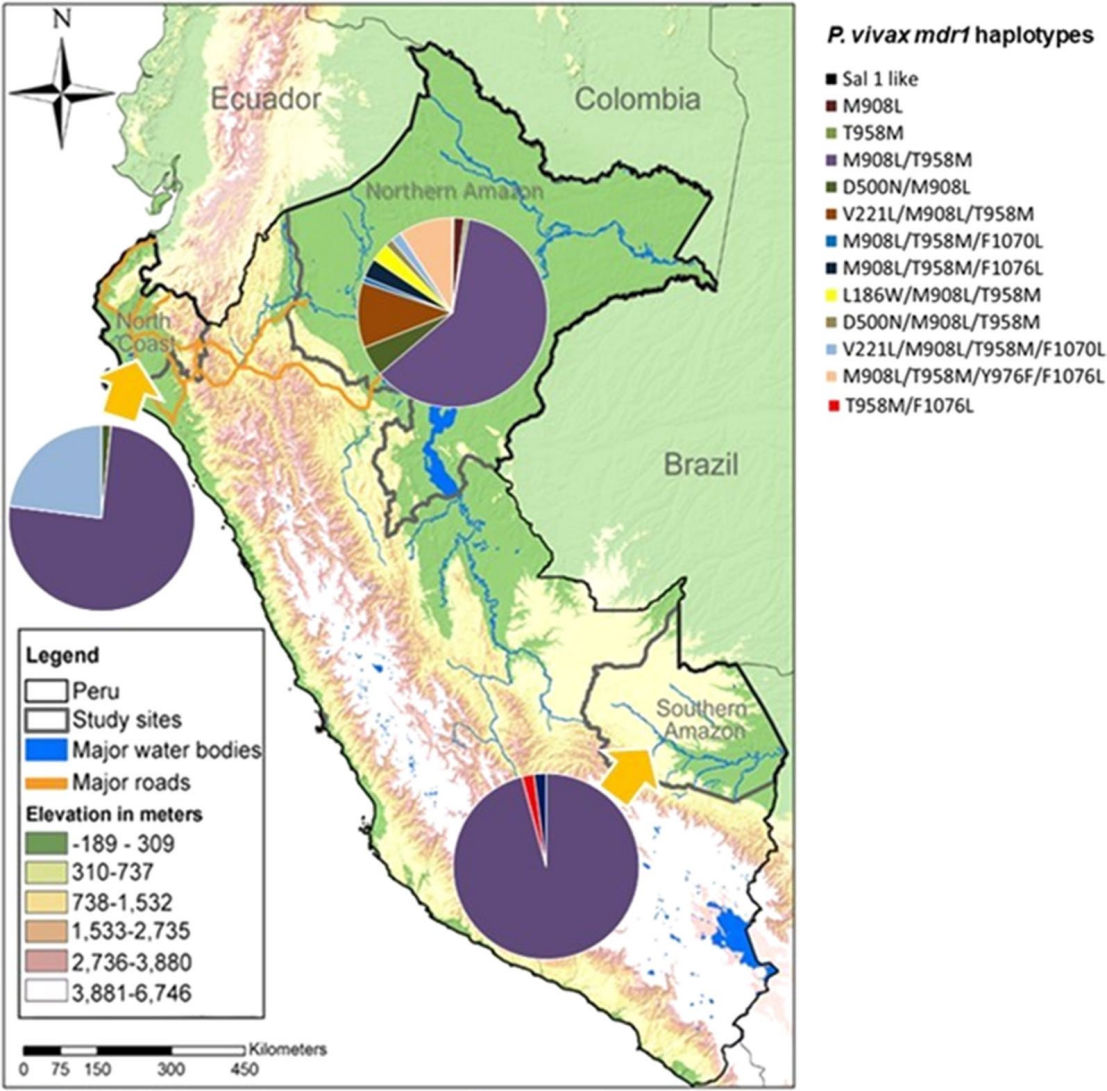
Geographic distribution of the *pvmdr1* haplotypes. The graphic was made using 550 samples collected in the Northern Amazon Basin (n = 445), the Southern Amazon Basin (n = 48) and the North Coast (n = 57). Pie charts indicate the *pvmdr1* haplotype present in a region and their prevalence. The M908L/T958M haplotype was distributed in all sites while the T958M/F1076L haplotype was specific for the North Coast

Samples were collected from four IRB-approved studies conducted in Peru between 2006 and 2015 in different communities of the Northern Amazon Basin (region of Loreto; 2006–2015), Southern Amazon Basin (region of Madre de Dios; 2012–2013) and North Coast (regions of Piura and Tumbes; 2008–2010) (Fig. 1). Although the Peruvian Amazon and the North Coast of Peru are different environments, they share common characteristics such as proximity to massive water sources like rivers and lakes and having a warm humid climate.

### Sample collection and preparation

Blood samples were collected by finger prick or venipuncture and thick and thin blood smears were prepared for malaria microscopy and read at the Naval Medical Research Unit 6 (NAMRU-6) facilities. DNA was extracted from either 200 µl venous blood or ~ 100 µl dried blood spots using the QIAamp® DNA Blood Mini Kit (GmbH, Hilden, Germany) following the manufacturer’s instructions and stored at − 20 °C. *Plasmodium vivax* monoinfection was confirmed by polymerase chain reaction of the 18S small subunit ribosomal RNA gene (18S SSU rRNA) as previously described [34].

### Identification of single nucleotide polymorphisms (SNP) in the *pvmdr1* gene

The whole *pvmdr1* gene was amplified by PCR in three overlapping fragments (Additional file 1: Fig. S1, Additional file 2: Table S1). PCR reaction was carried out in a 50 µl reaction volume containing 5 µl of gDNA (~ 25 ng), 1X buffer, 2 mM MgCL_2_, 125 µM dNTP’s, 250 nM of each primer and 1 unit of Taq Polymerase (Invitrogen). PCR products were purified using QIAquick™ silica‐based spin columns (GmbH, Hilden, Germany) and 30 ng of purified PCR product was used to amplify in a standard reaction (20 µl) of BigDye™ Terminator v3.1. The resulting products were sequenced on an ABI3130xl (Applied Biosystems) and analysed in the program Sequencher 4.1 (Gen Codes Corporation) using the reference Sal I strain of *P. vivax* (Salvador I, GenBank accession number AY571984).

### Data analysis

Data was entered in Microsoft Excel and analysed in STATA 13.0 for Windows. Absolute and relative frequency of mutant and wild type alleles and haplotypes of the *pvmdr1* gene were calculated for each region. Chi square and 2-tailed Fisher’s exact tests were used to assess statistical significant differences in proportions according to geographic regions. Nucleotide diversity, Tajima´s D test and F_*ST*_ values were calculated in dnaSP v5.1 [28, 35]. In addition, Mega v7.0 was used to assess for natural selection using the modified Nei-Gojobori method [36]. Finally, a haplotype network was constructed in PopArt 1.7 [37] to assess the relatedness of all isolates based on their *pvmdr1* sequences.

## Results

### Polymorphisms on the *pvmdr1* gene

The *pvmdr1* gene was sequenced on 550 *Plasmodium vivax* samples with no mixed genotypes from the three study areas. The majority of sequenced samples were collected from the Northern Amazon Basin (n = 445, 80.9%) followed by the Northern Coast (n = 57, 10.4%) and the Southern Amazon basin (n = 48, 8.7%).

**Fig. 2:**
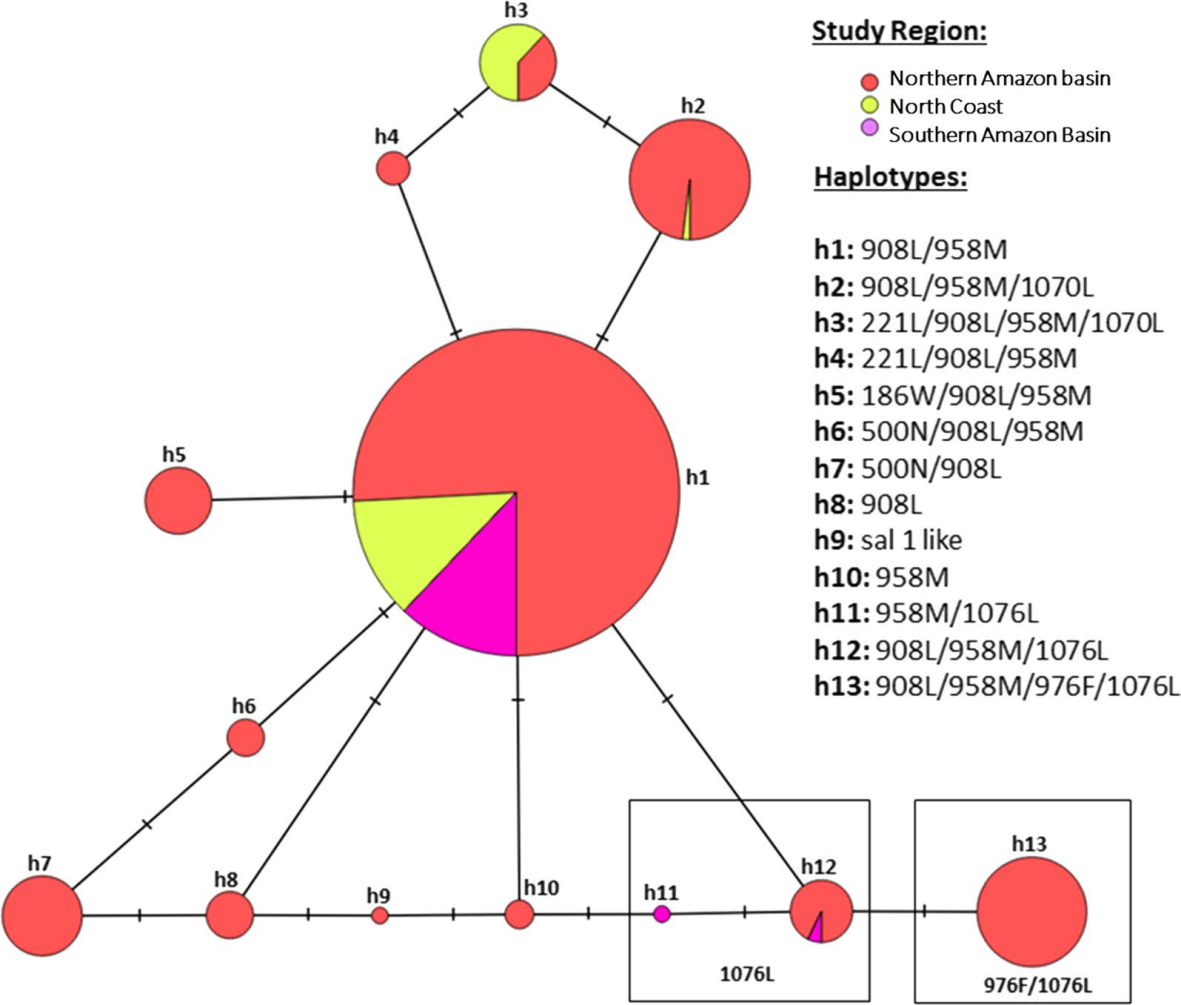
Haplotype network for *P. vivax mdr1* using 8 non-synonymous mutations for the study areas of Peru (n = 550). Each circle represent an independent haplotype, the lines connect nearby haplotypes and the cross line represent one non-synonymous mutation

A total of eleven mutations were found in *pvmdr1* (eight non-synonymous and three synonymous mutations) (Additional file 3: Table S3). The non-synonymous mutations were L186W (2.5%), V221L (12.4%), D500N (5.5%), M908L (99.1%), T958M (94.0%), Y976F (7.5%), F1070L (4.0%) and F1076L (10.5%). From all these mutations, statistical significant differences on the prevalence of SNPs across regions were found on five polymorphisms (Fig. 2 and Table 1).

**Table 1:** Prevalence of nonsynonymous mutations by regions 2006–2015

SNPs	Regions						p-value^a^	Total (n = 550)	
	Northern Amazon Basin (n = 445)		Southern Amazon Basin (n = 48)		North Coast (n = 57)				
	n	%	n	%	n	%		n	%
L186W (TTG–> TGG)	14	3.1	0	0.0	0	0.0	0.269	14	2.5
V221L (GTG–> CTG)^b^	58	13.0	0	0.0	10	17.5	0.001	68	12.4
D500N (GAT–> AAT)	29	6.5	0	0.0	1	1.8	0.062	30	5.5
M908L (ATG–> CTG)	441	99.1	47	97.9	57	100.0	0.437	545	99.1
T958M (ACG–> ATG)^b^	413	92.8	48	100.0	56	98.2	0.039	517	94.0
Y976F (TAC–> TTC)^b^	41	9.2	0	0.0	0	0.0	0.001	41	7.5
F1070L (TTC–> CTC)^b^	12	2.7	0	0.0	10	17.5	0.001	22	4.0
F1076L (TTT–> CTT)^b^	56	12.6	2	4.2	0	0.0	0.001	58	10.5

^a^Chi square test to compare SNPs frequency across regions

^b^Mutations with statistical significant differences across regions

The Y976F and F1076L mutations that are associated with CQ resistance were present in the Northern Amazon Basin in the 908L/958 M/976F/1076L and 908L/958 M/1076L haplotypes (Y976F + F1076L = 9.2%, F1076L = 3.4%) and in the 908L/958 M/1076L and 958 M/1076L haplotypes from the Southern Amazon Basin (F1076L = 2.1%) (Additional file 3: Table S3). Synonymous mutations on *pvmdr1* were found at positions T529 (ACA > ACG), L1022 (CTA > TTA) and K1355 (AAA > AAG), which were present in 57.8%, 18.0% and 3.4% of the samples, respectively.

**Fig. 3:**
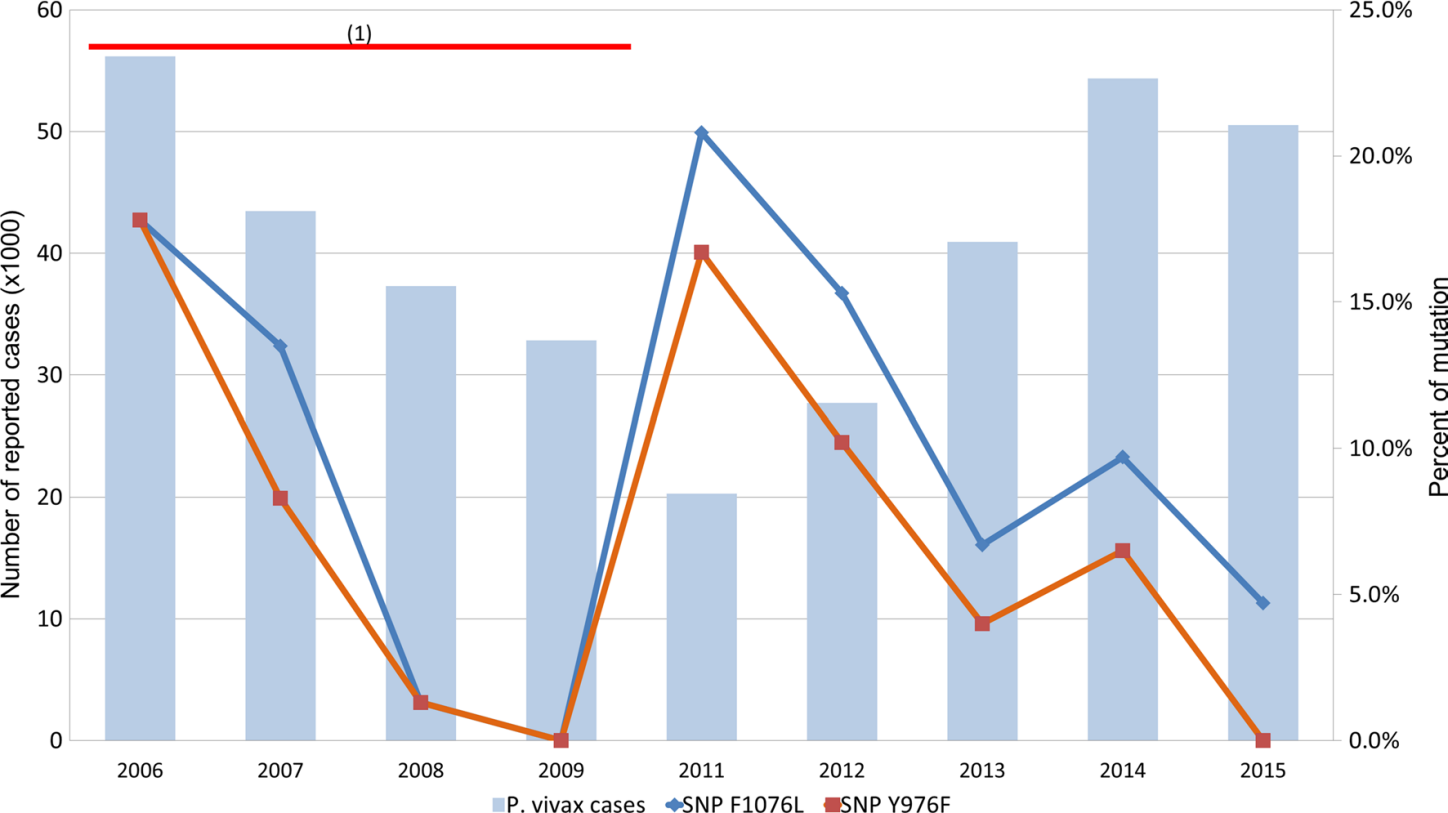
Fluctuation of non-synonymous mutations in the Northern Amazon Basin between 2006 and 2015. The graphic shows the dynamics of the 976F and 1076L polymorphisms and the number of reported cases in the Northern Amazon Basin. The “y” axis on the left shows the number of reported cases for the barplot. The “y” axis on the right depicts the percentage of the 976F and 1076L mutations for the lineplot whereas the “x” axis indicate the years. (1) Global fund’s Malaria project “PAMAFRO” (2005–2010). *Plasmodium vivax* data from 2010 was not included in the graphic because of the low sample size for that year (n = 3)

The frequencies of the Y976F and F1076L mutations fluctuated over time during the course of the study (Fig. 3). In this regard, there was a continuing decrease in the distribution of both mutations from 17.8% in 2006 to less than 2% in 2009. The highest prevalence for both mutations was recorded in 2011 (Y976F = 16.7% and F1076L = 20.8%) followed by a downward trend until 2013 (Y976F = 4% and F1076L = 6.7%).

### Geographic distribution of *pvmdr1* haplotypes

Twenty-seven haplotypes consisting of either synonymous and non-synonymous mutations were identified in *pvmdr1* (Additional file 3: Table S3). Out of those, 13 haplotypes consisting of non-synonymous mutation were identified on *pvmdr1* with all haplotypes but one presenting at least one non-synonymous mutation (Table 2).

**Table 2:** Prevalence of haplotypes by regions 2006–2015

Haplotypes	Regions						Total (n = 550)	
	Northern Amazon Basin (n = 445)		Southern Amazon Basin (n = 48)		North Coast (n = 57)			
	n	%	n	%	n	%	n	%
Sal 1 like	1	0.2	0	0.0	0	0.0	1	0.2
908L	8	1.8	0	0.0	0	0.0	8	1.5
958 M	3	0.7	0	0.0	0	0.0	3	0.6
958 M/1076L	0	0.0	1	2.1	0	0.0	1	0.2
908L/958 M^a^	272	61.1	46	95.8	46	80.7	364	66.2
500 N/908L	23	5.2	0	0.0	1	1.8	24	4.4
221L/908L/958 M	50	11.2	0	0.0	0	0.0	50	9.1
908L/958 M/1070L	4	0.9	0	0.0	0	0.0	4	0.7
908L/958 M/1076L	15	3.4	1	2.1	0	0.0	16	2.9
186 W/908L/958 M	14	3.1	0	0.0	0	0.0	14	2.6
500 N/908L/958 M	6	1.3	0	0.0	0	0.0	6	1.1
221L/908L/958 M/1070L	8	1.8	0	0.0	10	17.5	18	1.5
908L/958 M/976F/1076L	41	9.2	0	0.0	0	0.0	41	7.5

^a^Haplotypes with statistical significant differences across regions

Significant differences were found in the distribution of the 908L/958M haplotype among the three sites (p < 0.05). The Northern Amazon Basin presented the highest number of different haplotypes (92.3%) followed by the Southern Amazon basin (23%) and the North Coast (23%) (Fig. 1, Table 2). A common haplotype that was differentially (p < 0.05) distributed in all sites was M908L/T958M, which accounted for 61.1% of cases in the Northern Amazon Basin, 95.8% in the Southern Amazon and 80.7% in the North coast.

Single mutation was observed in 11 isolates (2%), double mutation in 389 (70.7%), triple mutation in 90 (16.2%) and quadruple mutation in 59 samples (10.7%). The F1076L mutation was present in three haplotypes (10.6% of the samples), while the combination Y976F/F1076L was present in one haplotype (908L/958M/976F/1076L) exclusive from the Northern Amazon Basin (7.5% of the samples).

The haplotype network of *pvmdr1* that comprised 8 non-synonymous mutations showed that all the haplotypes were closely related with most of them being only one mutational step from each other (Fig. 2).

### Genetic diversity of the *pvmdr1* locus

The overall nucleotide diversity of *pvmdr1* in Peru was π = 0.00054 with differences according to geographical location. In this regard, the Northern Amazon Basin was the most diverse (π = 0.00055) followed by the Southern Amazon basin (π = 0.00035) and the North coast (π = 0.00014).

F_*ST*_ values were high across all regions: 0.399 between the North coast and the Southern Amazon basin, 0.227 between North Coast and the Northern Amazon Basin, and 0.316 between the Southern Amazon basin and the Northern Amazon Basin. Moreover, Tajima´s D test was positive (1.321) although not statistically significant when samples were analyzed globally. At the regional level, Tajima’s D was also not significant across all regions, the Northern Amazon Basin (2.072), Southern Amazon basin (0.925) and North coast (− 0.612). This is also supported by a non-significant d_N_-d_S_ ratio for Peru (− 0.709; *P* = 1.000) and at the regional level: the Northern Amazon Basin (− 0.720; *P* = 1.000), the North Coast (− 1.106; *P* = 1.000) and Southern Amazon Basin (1.587; *P* = 0.058).

## Discussion

Different studies indicate that *P. vivax* has recently suffered an important evolutionary pressure driven by the use of antifolate drugs [38, 39]. Furthermore, in areas with *P. vivax* CQ susceptibility and *P. falciparum* CQ resistance, *P. vivax* is subjected to indirect selection pressures during the treatment of *P. falciparum* or mixed infections [40].

Although most mutations in *pvmdr1* play no role in parasite resistance, the Y976F and F1076L mutations are still controversial in the scientific community due to conflicting evidence [17, 18, 21, 22, 24, 41]. For instance, Thailand, whose anti-malarial treatment regime for *P. vivax* is based in CQ and PQ as in Peru [42], presented a frequency of 25% of the Y976F mutation [22], 21% of *pvmdr1* amplifications [29], CQ susceptibility and reduced susceptibility to MQ and ART in *P. vivax* [43]

In contrast, countries such as Indonesia or New Guinea showed up to 95% of the 976F mutation in *pvmdr1*, no evidence of *pvmdr1* amplification, CQ resistance and susceptibility to MQ and ART [29]. Furthermore, the Y976F mutation was associated with a fourfold higher chloroquine IC50 and 5 to 8 folds lower IC50 for ART and MQ, respectively [22]. A similar situation has been reported in French Guyana that presented a decrease in *pvmdr1* amplification from 71.3% when MQ was used against *P. falciparum* (1995–2002) to 12.8% after a change in the anti-malarial regime. This change was accompanied by an increase in 976F haplotypes after the use of MQ [40]. Unfortunately, the present study was not able to assess changes in *pvmdr1* copy number and evaluate if there was a relation between 976F haplotypes and *pvmdr1* amplification.

In the case of Peru, it appears that drug pressure and changes in the incidence of malaria have also affected the prevalence of the Y976F and F1076L genotypes in the Northern Amazon Basin, which decreased from 17.8% in 2006 to 1.2% in 2008. This decrease coincides with the implementation of a radical *P. vivax* treatment scheme in 2005 in the Northern Peruvian Amazon, which changed from 3 days of CQ and 14 days of PQ (0.25 mg/kg/day) to 3 days of CQ and 7 days of PQ (0.5 mg/kg/day) [2, 44]. Therefore, it is highly likely that the new treatment scheme coupled with increased access to diagnosis and treatment accessibility provided by PAMAFRO could have exerted strong selection pressures in the *P. vivax* population. This is further supported by the fact that Peru reached a malaria incidence rate lower than 1 case/1,000 inhabitants towards the end of PAMAFRO in 2010 [2]. However, after the end of PAMAFRO, the frequency of the Y976F and F1076L genotypes radically increased until reaching 20.83% in 2011 to initiate a gradual reduction down to 5% in 2015. This rapid fluctuation in the frequencies of these genotypes could be due to local variability in malaria control activities rather than reversion of the mutated genotype.

The 908L/958M haplotype that was the most frequent in our study presented a continuous increase in the Northern Amazon Basin from 48% in 2006 to 71% in 2015. No major changes in the frequencies of the other haplotypes were found. In this regard, studies carried out in *P. falciparum* suggest that CQ-wildtype alleles in *pfmdr1* and *pfcrt* have a selective advantage over resistant genotypes when CQ pressure is not exerted [45–47]. In this regard, regions where CQ was discontinued experienced an increase of wildtype *pfcrt* and *pfmdr1 P. falciparum* strains [45, 46, 48]. However, this is unlikely to happen with the *P. vivax* population in the Peruvian Amazon due to the continued use of CQ as first-line treatment. Therefore, it is possible that the changes in the prevalence of *pvmdr1* genotypes correspond to expansion or contraction of the wildtype parasite population in response to local and temporal variations of malaria control.

The M908L and T958M mutations that are two of the most frequent polymorphisms in our study (99.1% and 94.0%, respectively) were reported with 100% prevalence in Thailand and Madagascar and with 28% and 100% prevalence in Brazil, respectively [9, 15, 24, 49]. Also, these studies suggested that these polymorphisms do not have any association with resistance to chloroquine and are likely to be related to the evolutionary history of *P. vivax* [9, 15, 24, 26, 27, 49].

In terms of diversity, the Northern Amazon Basin presented 12 of the 13 haplotypes found in Peru being the region with the highest diversity for *pvmdr1* (π = 0.00055) compared to the global value of *pvmdr1* in Peru (π = 0.00054) [50]. This level of diversity is lower to the ones from other endemic areas such as India (π = 0.0012), Brazil (π = 0.0016) and Ecuador (π = 0.0009) [28]. In this regard, the low diversity in Peru and Ecuador could be explained by frequent inbreeding and low recombination rates which are characteristic of low transmission regions in contrast to the high recombination rates between *P. vivax* genotypes in India and Brazil [39, 51, 52].

The North Coast (π = 0.00014) and Southern Amazon Basin (π = 0.00035) had a lower diversity which is consistent with previous studies in the North Coast that showed a low parasite diversity and a homogeneous population structure [52]. This is likely due to the presence of the Andes mountain range that acts as a geographical barrier that blocks migration of infected Amazonian vectors. Another factor that could influence the low parasite diversity in the North Coast is the drastic fluctuation of malaria prevalence over time due to the El Niño Southern Oscillation, the presence of a different malaria vector than the one in the Peruvian Amazon basin and to the low human migration between both sites [2].

In the case of the Southern Amazon basin, distribution and transmission of vector-borne diseases including malaria has suffered rapid fluctuations during the last decade due to illegal mining, logging and agriculture, which have drastically changed the environment [2, 53]. In addition, it is also possible that differences in diversity could be explained by the genetic background of circulating populations in the distinct regions of Peru.

However, it is important to consider that a single marker and a potential target under selective pressure might have low resolution to accurately estimate genetic diversity. This is particularly important in low transmission regions such as Peru where there is already strong evidence of low genetic diversity. Therefore, further studies targeting multiple markers or using next generation sequencing are needed to confirm our results for *pvmdr1*.

Finally, although there is a strong association between molecular markers and resistance to anti-malarials *in P. falciparum*, this relationship is not clear for *P. vivax* and its orthologs [22, 28, 47]. This demands further research to assess their effect at the clinical level in order to confirm variants associated with resistance and therapeutic failure. Furthermore, continuous monitoring of *pvmdr1* together with in vitro susceptibility tests would help to assess changes in the transmission of malaria overtime as a result of human and environmental variables to support initiatives for malaria control and elimination.

## Conclusion

This research showed a regional diversification of *pvmdr1* across endemic malaria regions in Peru and changes over time in the frequency of *pvmdr1* genotypes in the Northern Amazon Basin between 2006 and 2015. This information is relevant for future epidemiological surveillance to measure the emergence of resistance and changes on the parasite population in this and other endemic areas. However, additional research is needed to elucidate the role of *pvmdr1* in *P. vivax* resistance complemented with ex vivo and phenotypic data.

## Supplementary information

**Additional file 1: Fig. S1.** Amplified and sequenced regions of the pvmdr1 gene.

**Additional file 2: Table S1. **Primers for conventional PCR, Nested PCR and sequencing ofpvmdr1 gene.

**Additional file 3: Table S3. **Prevalence of haplotypes consisting of synonymous and nonsynonymousmutation.

## Data Availability

All data generated and/or analyzed during this study are included in this published article and its additional files.

## Abbreviations

IRB: Institutional review board
PAMAFRO: Global Fund Malaria Project
PCR: Polymerase chain reaction
CQ: Chloroquine
PQ: Primaquine
ART: Artesunate
SP: Sulfadoxine-pyrimethamine
MQ: Mefloquine
