# Supplementary figures and images for: Molecular surveillance of the Plasmodium vivax multidrug resistance 1 gene in Peru between 2006 and 2015
Source: Malar J. 2020 Dec 4;19:450. doi: 10.1186/s12936-020-03519-8 (PMC7718670; doi:10.1186/s12936-020-03519-8)

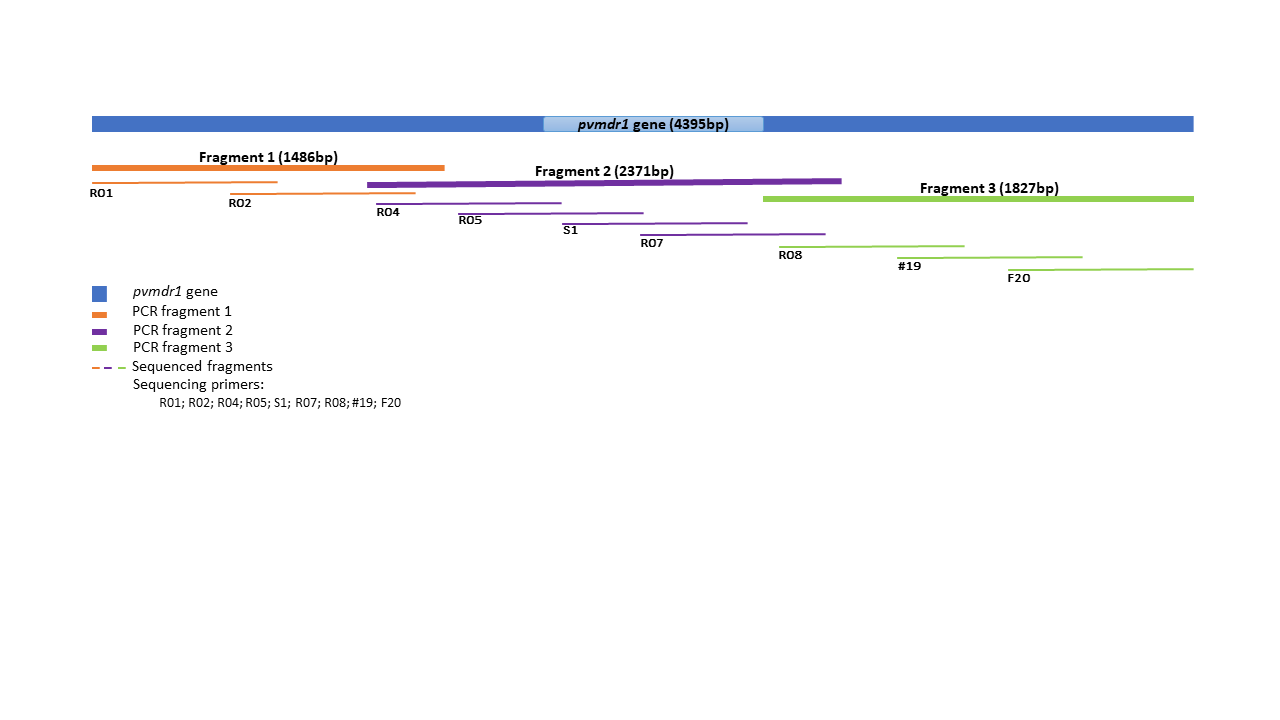

Supplement: Supplementary file 1 — Additional file 1: Fig. S1. Amplified and sequenced regions of the pvmdr1 gene. [file 12936_2020_3519_MOESM1_ESM.tif]
