# Supplementary material for: Molecular surveillance of the Plasmodium vivax multidrug resistance 1 gene in Peru between 2006 and 2015
Source: Malar J. 2020 Dec 4;19:450. doi: 10.1186/s12936-020-03519-8 (PMC7718670; doi:10.1186/s12936-020-03519-8)
Supplement: Supplementary file 2 — Additional file 2: Table S1. Primers for conventional PCR, Nested PCR and sequencing ofpvmdr1 gene. [file 12936_2020_3519_MOESM2_ESM.docx]

**Additional file 2: Table S1: Primers for conventional PCR, Nested PCR and sequencing of *pvmdr1* gene**

|  |  | **Primer sequence 5' - 3'** | **Size amplicon (bp)** |
| --- | --- | --- | --- |
| **conventional PCR** | **Fragment 1** |  |  |
|  | *Pv#11* | ATGAAAAAGGATCAAAGGCAAC | 1486 |
|  | *PvR03* | CCTCCGATAAGGCTTCTAAAT |  |
|  | **Fragment 2** |  |  |
|  | *pvmdr13* | CATCTCCATCCTTCTAG | 2371 |
|  | *pvmdr17* | GGCGTAGCTTCCCGTA |  |
|  | **Fragment 3** |  |  |
|  | *PvF07* | GCAAACCTGGAGGCGAACT | 1827 |
|  | *Pv#20* | CTACTTAGCCAGCTTGACGTACTTC |  |
|  |  |  |  |
| **Nested PCR*** | **Fragment 1** |  |  |
|  | *Pv#11* | ATGAAAAAGGATCAAAGGCAACCC | 1233 |
|  | *R02* | TCCTTCCGTGAGGGTAAAA |  |
|  | **Fragment 2** |  |  |
|  | *pvmdr13_mod* | GTCATCTCCATCCTTCTAGGA | 2257 |
|  | *PvR07* | ATTGGGCACTCTGACTGAAC |  |
|  | **Fragment 3** |  |  |
|  | *PvF08* | CGAGTCAACCAACCAGGCACA | 1401 |
|  | *Pv#20* | CTACTTAGCCAGCTTGACGTACTTC |  |
|  |  |  |  |
| **Sequencing** | **Fragment 1** |  |  |
|  | *R01* | ACGTGATGCAGAGGGTGA |  |
|  | *R02* | TCCTTCCGTGAGGGTAAAA |  |
|  | **Fragment 2** |  |  |
|  | *R04* | TGGTGCTCAGCCTGTGGG |  |
|  | *R05* | TTGCTTTGGCGTCACTCA |  |
|  | *S1* | CGCTAGAGCCATTATTAGAAACCCC |  |
|  | *R07* | ATTGGGCACTCTGACTGAAC |  |
|  | **fragment 3** |  |  |
|  | *R08* | AATCGTAGAATCGCATCAGC |  |
|  | *#19* | TGACGTCCTCTCTGGTGGCATT |  |
|  | *F20* | CTACTTAGCCAGCTTGACGTAC |  |
| *Nested PCR was only used in samples with low parasitaemia. | | | |
