# Supplementary material for: Molecular surveillance of the Plasmodium vivax multidrug resistance 1 gene in Peru between 2006 and 2015
Source: Malar J. 2020 Dec 4;19:450. doi: 10.1186/s12936-020-03519-8 (PMC7718670; doi:10.1186/s12936-020-03519-8)
Supplement: Supplementary file 3 — Additional file 3: Table S3. Prevalence of haplotypes consisting of synonymous and nonsynonymousmutation. [file 12936_2020_3519_MOESM3_ESM.docx]

| **Additional file 3: Table S2: Prevalence of haplotypes consisting of synonymous and non-synonymous mutation** | | | | |
| --- | --- | --- | --- | --- |
|  |  |  |  |  |
| **Haplotypes** | **Regions** | | | **Total n=550 (%)** |
|  | **Loreto (%)** | **North Coast (%)** | **Southern Amazon (%)** |  |
| 529T(AC**G**)/1022L(CTA)/1355K(AAA) | 100.0 | 0.0 | 0.0 | 1 (0.18) |
| 529T(AC**G**)/908L/1022L(CTA)/1355K(AAA) | 100.0 | 0.0 | 0.0 | 6 (1.09) |
| 529T(ACA)/908L/1022L(CTA)/1355K(AAA) | 100.0 | 0.0 | 0.0 | 2 (0.36) |
| 529T(AC**G**)/958M/1022L(CTA)/1355K(AAA) | 100.0 | 0.0 | 0.0 | 1 (0.18) |
| 529T(ACA)/958M/1022L(CTA)/1355K(AAA) | 100.0 | 0.0 | 0.0 | 2 (0.36) |
| 529T(AC**G**)/958M/1022L(CTA)/1076L/1355K(AA**G**) | 0.0 | 0.0 | 100.0 | 1 (0.18) |
| 529T(ACA)/908L/958M/1022L(CTA)/1355K(AAA) | 15.6 | 42.2 | 42.2 | 109 (19.82) |
| 529T(ACA)/908L/958M/1022L(**T**TA)/1355K(AAA) | 100.0 | 0.0 | 0.0 | 26 (4.73) |
| 529T(AC**G**)/908L/958M/1022L(CTA)/1355K(AAA) | 100.0 | 0.0 | 0.0 | 224 (40.73) |
| 529T(AC**G**)/908L/958M/1022L(**T**TA)/1355K(AAA) | 100.0 | 0.0 | 0.0 | 5 (0.91) |
| 500N/529T(AC**G**)/908L/1022L(CTA)/1355K(AAA) | 95.5 | 4.5 | 0.0 | 22 (4) |
| 500N/529T(ACA)/908L/1022L(CTA)/1355K(AAA) | 100.0 | 0.0 | 0.0 | 2 (0.36) |
| 221L/529T(ACA)/908L/958M/1022L(CTA)/1355K(AAA) | 100.0 | 0.0 | 0.0 | 39 (7.09) |
| 221L/529T(AC**G**)/908L/958M/1022L(CTA)/1355K(AAA) | 100.0 | 0.0 | 0.0 | 11 (2) |
| 529T(AC**G**)/908L/958M/1022L(**T**TA)/1070L/1355K(AA**G**) | 100.0 | 0.0 | 0.0 | 4 (0.73) |
| 529T(ACA)/908L/958M/1022L(CTA)/1076L/1355K(AAA) | 100.0 | 0.0 | 0.0 | 3 (0.55) |
| 529T(ACA)/908L/958M/1022L(CTA)/1076L/1355K(AA**G**) | 87.5 | 0.0 | 12.5 | 8 (1.45) |
| 529T(ACA)/908L/958M/1022L(**T**TA)/1076L/1355K(AAA) | 100.0 | 0.0 | 0.0 | 1 (0.18) |
| 529T(AC**G**)/908L/958M/1022L(CTA)/1076L/1355K(AAA) | 100.0 | 0.0 | 0.0 | 1 (0.18) |
| 529T(AC**G**)/908L/958M/1022L(**T**TA)/1076L/1355K(AAA) | 100.0 | 0.0 | 0.0 | 3 (0.55) |
| 186W/529T(AC**G**)/908L/958M/1022L(CTA)/1355K(AAA) | 100.0 | 0.0 | 0.0 | 14 (2.55) |
| 500N/529T(AC**G**)/908L/958M/1022L(CTA)/1355K(AAA) | 100.0 | 0.0 | 0.0 | 5 (0.91) |
| 500N/529T(AC**G**)/908L/958M/1022L(**T**TA)/1355K(AAA) | 100.0 | 0.0 | 0.0 | 1 (0.18) |
| 221L/529T(AC**G**)/908L/958M/1022L(**T**TA)/1070L/1355K(AAA) | 41.7 | 58.3 | 0.0 | 12 (2.18) |
| 221L/529T(AC**G**)/908L/958M/1022L(**T**TA)/1070L/1355K(AA**G**) | 50.0 | 50.0 | 0.0 | 6 (1.09) |
| 529T(ACA)/908L/958M/976F/1022L(**T**TA)/1076L/1355K(AAA) | 100.0 | 0.0 | 0.0 | 40 (7.27) |
| 529T(AC**G**)/908L/958M/976F/1022L(**T**TA)/1076L/1355K(AAA) | 100.0 | 0.0 | 0.0 | 1 (0.18) |
| () synonymous mutation | | | | |
